# The different axes of the mammalian mitochondrial unfolded protein response

**DOI:** 10.1186/s12915-018-0548-x

**Published:** 2018-07-26

**Authors:** Christian Münch

**Affiliations:** Institute of Biochemistry II, Goethe University – Medical Faculty, University Hospital, Frankfurt am Main, Germany

## Abstract

Mitochondria are sensitive to numerous environmental stresses, which can lead to activation of mitochondrial stress responses (MSRs). Of particular recent interest has been the mitochondrial unfolded protein response (UPR^mt^), activated to restore protein homeostasis (proteostasis) upon mitochondrial protein misfolding. Several axes of the UPR^mt^ have been described, creating some confusion as to the nature of the different responses. While distinct molecularly, these different axes are likely mutually beneficial and activated in parallel. This review aims at describing and distinguishing the different mammalian MSR/UPR^mt^ axes to define key processes and members and to examine the involvement of protein misfolding.

## Mitochondrial protein folding

Mitochondria are highly regulated cellular organelles that fulfill numerous metabolic functions, including the production of ATP by respiration. Function and quality of mitochondria need to be tightly controlled to ensure the supply of metabolic building blocks and to prevent the production of harmful agents such as reactive oxygen species (ROS), produced at increased rates upon malfunctioning respiration [1, 2]. Mitochondrial aging, environmental changes such as fever and medication, and numerous pathologies including cancer, Alzheimer’s disease, Parkinson’s disease, and amyotrophic lateral sclerosis involve mitochondrial dysfunction [3–7]. It is crucial to understand the mitochondrial responses elicited upon these conditions to recognize underlying mechanisms. Indeed, for several mitochondrial stresses, such as hypoxia and oxidative stress, the resulting stress responses are well understood, including the pathways by which they potentially trigger cell death [8, 9]. However, how cells react to perturbation in mitochondrial proteostasis caused by accumulation of misfolded proteins is still unclear, despite the significant impact of protein aggregation on mitochondrial function and cellular health.

Mitochondria are cellular organelles separated from the extra-mitochondrial environment by two membranes—the outer mitochondrial membrane (OMM) and the inner mitochondrial membrane (IMM). The compartment enclosed by the IMM is called the matrix, and the space between the OMM and IMM defines the intermembrane space (IMS). Due to the archetypic origin of mitochondria and the resulting physical separation from the cytosol, the mitochondrial matrix forms a largely independent protein compartment providing its own translation and protein quality control machinery including chaperones and proteases [1, 10–12]. Mitochondria are composed of well over 1000 proteins, the majority located in the matrix [13]. Most of these proteins are encoded in the nuclear genome and imported into mitochondria [14]. Thirteen transmembrane proteins of the respiratory chain are encoded in the mitochondrial genome (mtDNA), together with a set of 22 tRNAs and two rRNAs, required for the assembly of a translation machinery in the matrix [15, 16]. Inside the matrix, both imported and mitochondrially translated proteins are folded and need to be quality controlled to maintain mitochondrial proteostasis [11, 15, 17]. Therefore, mitochondria contain their own set of matrix localized heat shock proteins (HSP) 70 and 90, chaperonins, and proteases.

**Fig. 1:**
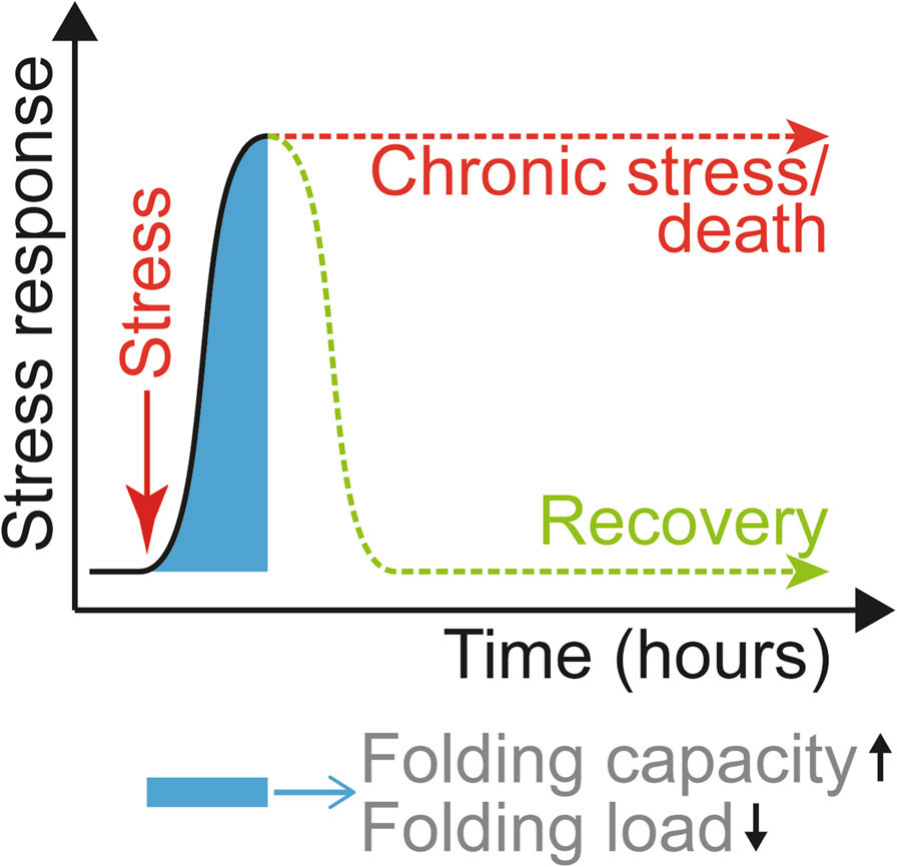
Folding stress responses. Protein misfolding activates transient, pro-survival stress responses that increase the folding capacity (i.e., modulation of chaperone and protease levels) and decrease the folding load (i.e., decrease in translation) to restore proteostasis. Responses typically last several hours. Prolonged stress activation that cannot alleviate the stress causes alternative outcomes, including cell death. Pharmacological induction of protein misfolding allows the study of the acute response to protein misfolding. Chronic activation of the stress, as observed upon genomic modulation or in disease, leads to the activation of alternative pathways and potentially cell death

**Fig. 2:**
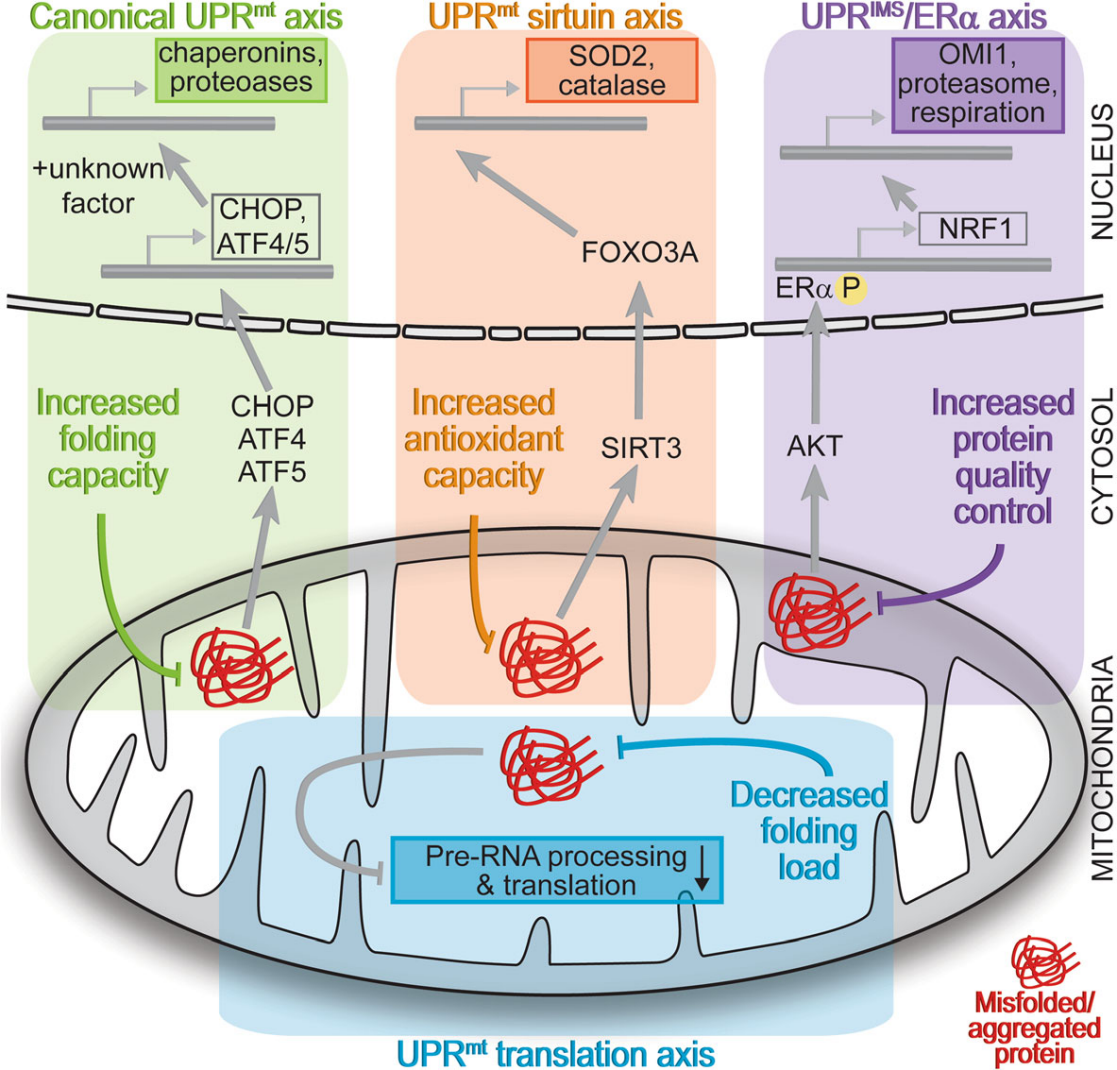
The different mammalian UPR^mt^ axes. Depiction of the different UPR^mt^ axes that are activated upon mitochondrial protein misfolding/aggregation: (1) The canonical UPR^mt^ leads to altered localization and levels of CHOP, ATF4, and ATF5. These, together with other unknown transcription factors, lead to the induction of the chaperonins, chaperones, and proteases to increase the folding capacity inside mitochondria. (2) SIRT3 becomes activated as part of the UPR^mt^ sirtuin axis leading to the deacetylation and relocalization of FOXO3A to the nucleus, where it induces SOD2 and catalase as part of an antioxidant response. (3) Protein misfolding in the intermembrane space activates the UPR^IMS^–ERα axis, which acts via AKT and ROS-dependent phosphorylation of ERα, causing induction of *NRF1*. This in turn leads to increased protease levels, modulation of respiration levels, and enhanced proteasome activity to increase the protein quality control capacity. (4) The UPR^mt^ translation axis is a local response, largely independent of transcriptional effects in the nucleus. Protein unfolding in the matrix causes the rapid degradation of components of the pre-RNA processing machinery and a shutdown of mitochondrial translation to decrease the mitochondrial folding load

The proper function of proteins and maintenance of proteostasis entails the tight control of protein folding, including co-translational and post-translational folding, maturation, and degradation of proteins [18–22]. These processes must be maintained in all distinct cellular compartments to function correctly [23]. Upon proteostasis failure, stress responses are rapidly activated—typically in a time-course of several hours—in an attempt to alleviate proteostasis defects by modulating the folding environment through modification of protein synthesis and the availability of folding helpers—chaperones (Fig. 1). A hallmark of these responses is that they are highly acute, pro-survival responses that aim to alleviate transient stresses to restore homeostasis and support cell survival. However, upon chronic activation, they typically shift towards pro-death responses [24]. Stress responses like the heat shock response in the cytosol and the unfolded protein response in the endoplasmic reticulum (UPR^ER^) have been extensively studied and reviewed [25, 26]. However, knowledge about the role, function, and regulation of a mitochondrial stress response to unfolded proteins is lagging behind and details are much more uncertain. Similar to the UPR^ER^ that elicits a multi-axis response mediated by several receptors and leading to different effects such as induction of pro-folding factors and inhibition of translation [26], the UPR^mt^ also appears to contain several axes with distinct molecular outcomes (Fig. 2). However, their underlying molecular mechanisms and components remain largely unknown. This review will provide insight into these different axes of the mammalian UPR^mt^.

## Discovery of the UPR^mt^

In 1996, the Hoogenraad laboratory discovered a stress response that is specific to mitochondrial protein misfolding and that was later named the UPR^mt^. They described the gene locus of the nuclear-encoded, mitochondria-localized chaperonins HSPD1 and HSPE1 (also known as HSP60 and HSP10) [27], to be controlled by a bi-directional promoter, which shows significantly increased activity upon loss of the mtDNA and heat shock [28, 29]. This was proof of a specific mitochondrial response to folding stress within the organelle and it was shown to depend on mitochondrial–nuclear communication to elicit a specific feedback to improve folding conditions in mitochondria, independent of general heat-shock responses.

Extensive studies into the UPR^mt^ in *Caenorhabditis elegans* have uncovered molecular mechanisms involved in signaling the mitochondrial stress to the nucleus, causing induction of a transcriptional response [4]. This response is largely driven by the release of peptides from mitochondria by the transporter HAF-1 and detection of these peptides in the cytosol, and by a dual-localized transcription factor—activating transcription factor associated with stress–1 (ATFS-1)—whose import into mitochondria is inhibited upon UPR^mt^, leading to its accumulation in the nucleus and activation of the transcriptional UPR^mt^ [30, 31]. The UPR^mt^ pathways in *C. elegans* have been comprehensively reviewed [4, 32], but to what extent these mechanisms are conserved in mammalian cells remains unclear.

## The canonical UPR^mt^ response

The transcriptional response to mitochondrial protein misfolding described above remains the best understood mammalian UPR^mt^ axis and forms the canonical UPR^mt^. Its outcome is the induction of genes increasing the folding capacity in mitochondria. Employing misfolding-prone deletion mutants of the mitochondrial protein ornithine transcarbamylase (OTCΔ) aided in defining the UPR^mt^ in mammalian cells and determined its role in response to protein misfolding [33]. Exogenously expressed OTCΔ misfolds and accumulates in the matrix, triggering the induction of the chaperonin promoter via c-Jun N-terminal kinase 2 [33, 34]. Chaperonin induction by OTCΔ is transient and reversible, thus showing hallmarks typical for misfolding stress responses [24, 33]. Analysis of the mitochondrial chaperonin promoter uncovered a transcription factor C/EBP homologous protein (CHOP) binding element essential for its activation during OTCΔ-induced UPR^mt^ [33]. CHOP is known to be part of the integrated stress response (ISR), which is activated by any of four different kinases to integrate various cellular stresses (amino acid deprivation, heme deficiency, ER protein misfolding, or viral infection, mediated by the eukaryotic initiation factor 2 alpha (EIF2A) kinases GCN2, HRI, PERK, or PKR, respectively). Kinase phosphorylation of EIF2A causes alternative initiation and an increase in the translation of activating transcription factor 4 (ATF4), which activates numerous genes including *CHOP* [35].

Despite the dependence on ISR factors, i.e., CHOP, UPR^mt^ signaling is highly specific, as documented by the fact that the OTCΔ-triggered activation of *CHOP* does not increase *BiP* transcript levels, an ER chaperone induced by UPR^ER^ in a process also mediated by the ISR [23, 33]. While activation of the chaperonin promoter by CHOP occurs in association with C/EBPβ [33, 36], increasing CHOP and C/EBPβ levels are not sufficient to induce the chaperonins, demonstrating the need for further activating factors [36]. Recently, ATF5, which is induced by CHOP and ATF4 [37, 38], was found to be involved in retrograde signaling of the UPR^mt^ to the nucleus in a function similar to ATFS-1 in *C. elegans* [39]. Additionally, further analysis of the chaperonin promoter revealed two additional promoter elements—mitochondrial unfolded protein response element (MURE) 1 and 2—in close proximity to the CHOP elements that likely play a role in the specificity of UPR^mt^ signaling [36]. Which transcription factors bind these sites and whether they are essential for UPR^mt^ signaling in cells have not been determined. Luciferase reporter assay testing of potentially UPR^mt^-regulated promoters pointed towards further genes possibly being activated by the UPR^mt^ [36], some of which could, however, not be confirmed by analysis of endogenous transcripts [40]. Recent analysis of changes in the transcriptome upon acute induction of the UPR^mt^ revealed that induction of the canonical UPR^mt^ leads to specific and extensive transcriptional rearrangements affecting a wide range of biological pathways, mainly involved in protein folding and cellular homeostasis [41]. Combined, these findings described the canonical UPR^mt^ response as an extensive transcriptional response in the nucleus, triggered by mitochondrial unfolded proteins inducing chaperonin transcription via a mechanism involving a CHOP element in the promoter region.

The central role of CHOP in UPR^mt^ signaling is surprising as CHOP is also a key member of the ISR, which is induced by various other stresses and also forms part of the UPR^ER^ [23]. Overexpression of OTCΔ in murine intestinal epithelial cells causes induction of protein kinase double-stranded RNA-dependent (PKR), as seen in virus-related ISR, suggesting a possible role of this ISR type in the UPR^mt^ [42]. However, knockdown of any of the four EIF2A kinases capable of eliciting an ISR has no effect on the induction of *CHOP* upon acute UPR^mt^ induction mediated by mitochondrial HSP90 inhibition [41]. This implies that EIF2A kinases, at least individually, are not required for a UPR^mt^-mediated *CHOP* induction. The distinct regulation of CHOP hints towards other factors playing important roles in shaping the transcriptional outcome of the UPR^mt^, possibly by binding to MURE1 and MURE2 sites. Due to the lack of understanding of co-regulating factors and the inherent non-specificity of CHOP, the induction of *CHOP* must not be used as a readout for the induction of the UPR^mt^. Indeed, numerous mitochondrial stresses that are not related to protein misfolding, but instead inhibit central functions like respiration, mitochondrial membrane potential, import, and translation, can quickly induce CHOP (via ATF4) without leading to chaperonin activation [41, 43]. Thus, although ATF4 and CHOP play important roles in the UPR^mt^, likely regulating hitherto unknown aspects of the modulation of cellular processes by the UPR^mt^, they alone are not sufficient to elicit the canonical UPR^mt^ transcriptional response, which likely depends on additional signaling factors.

The following conditions causing mitochondrial protein misfolding have been shown to subsequently elicit the canonical UPR^mt^ defined by chaperonin induction: (1) overexpression of misfolding-prone deletion mutants of OTCΔ; (2) inhibition of mitochondrial HSP90 [41, 44, 45]; (3) inhibition of lon peptidase 1 (LONP1) [41, 46], which is crucial for the digestion of misfolded matrix proteins [12]; and (4) expression of another misfolding mitochondrial protein (EndoG, see below) [47, 48]. Due to the high chaperonin protein levels under basal conditions [49], the analysis of changes in their levels has proven difficult as a readout for the UPR^mt^ in mammalian cells, unless the UPR^mt^ is induced chronically, or highly quantitative methods such as mass spectrometry are used [41]. However, this issue is overcome by analyzing chaperonin transcript levels, which provide a robust increase, despite their high abundance in cells [41], and are now widely accepted as the gold standard marker for activation of the canonical UPR^mt^ axis, as documented by numerous publications [39, 41, 48, 50]. Induction of chaperonins exemplifies the role of the canonical UPR^mt^ to increase the mitochondrial folding capacity in response to protein misfolding; however, this is not the only mitochondrial response to protein misfolding.

## The UPR^mt^ translation axis

In addition to increasing folding capacity through induction of chaperones—the canonical UPR^mt^ transcriptional response—cells employ a second mechanism of decreasing the unfolded protein load, e.g., achieved by a reduced uptake of proteins into the organelle (for protein-importing compartments) or by reduced translation (for compartments containing a translation machinery). While work in *C. elegans* has shown a decrease in mitochondrial protein import and translation [31, 51], the effects of mitochondrial protein misfolding on import and translation in mammalian cells, and thus a role of the second principle, are not clear. Recently, J. Wade Harper and myself have provided the first evidence to support the existence of a mammalian UPR^mt^ that reduces the folding load: taking advantage of two inhibitors targeting the mitochondrial HSP90 and LONP1 [45, 46] to acutely induce the UPR^mt^, we discovered a translational UPR^mt^ axis that controls the folding load within mitochondria upon UPR^mt^ activation [41]. Whole strands of mtDNA are transcribed into long, polycistronic pre-RNAs that are processed by the RNase P complex, consisting of MRPP1–3 [15, 52, 53]. Upon acute induction of the UPR^mt^, we observed a rapid decrease of MRPP3 transcript and protein levels, causing a markedly lower level of mitochondrial pre-RNA processing and ultimately a reversible reduction in mitochondrial translation [41].

**Fig. 3:**
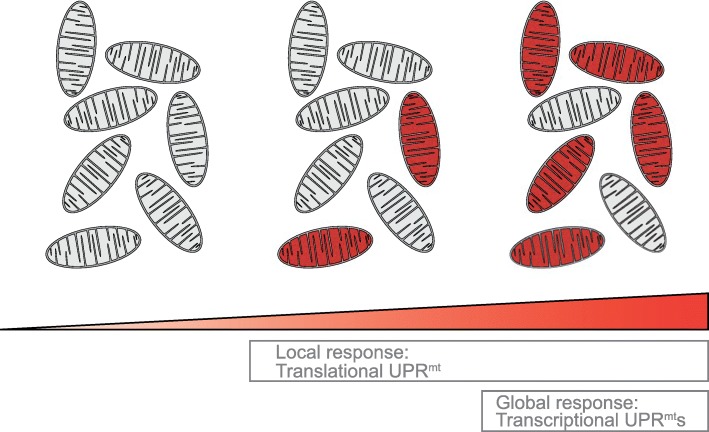
Integration of mitochondrial misfolding stress and different UPR^mt^ axes. Cells contain numerous mitochondria with a certain, low percentage stressed upon basal conditions, due to aging and metabolic damage. Dealing with these refined incidents of mitochondrial proteostasis defects, which are not due to significant environmental perturbation, requires spatially defined responses. The UPR^mt^ translational response acts via local, posttranslational regulation of MRPP3 levels and can decrease translation, and thus folding load, in an individual mitochondrion. Thus, it acts locally as a first response to mitochondrial protein misfolding in few damaged mitochondria without causing global effects. The transcriptional UPR^mt^ effects occur cell-wide and likely require passing a certain threshold of mitochondrial proteostasis defects. This suggests a model in which mitochondrion-specific UPR^mt^ effects (i.e., the UPR^mt^ translation axis) are activated upon cellular conditions with a certain low percentage of mitochondria suffering from protein misfolding and activation of the cell-wide UPR^mt^ axes upon proteostasis defects in a large percentage of mitochondria

This translational UPR^mt^ axis, which limits the protein folding load by regulating mitochondrial translation, constitutes an interesting new aspect to the UPR^mt^: due to its post-translational regulation within a single mitochondrion, there is no requirement to pass a cellular signaling threshold for activating the transcriptional UPR^mt^. Instead, it acts locally in single, damaged mitochondria and could thus form a first line of defense against mitochondrial damage that is most likely independent of extra-mitochondrial stimuli (Fig. 3): under cellular conditions with few stressed mitochondria, only the locally acting UPR^mt^ translation axis becomes activated to rapidly improve proteostasis without cell-wide effects. However, once a certain, larger number of mitochondria show perturbed proteostasis, reflective of possible harmful environmental conditions, the other UPR^mt^ axes are initiated to modulate the cellular proteome via global transcriptional rearrangements. Defects in pre-RNA processing are the cause of several human diseases [54–56]. Also, work in yeast and mouse hepatocytes has shown cellular programs to control the mito-nuclear protein balance, particularly with respect to subunits of the respiratory chain [57, 58], indicating that mitochondrial protein translation may have impacts on the cell. Future work will be required to understand the relationship between these diseases, pre-RNA processing, and the UPR^mt^. The translational UPR^mt^ axis described here is highly complementary to the transcriptional canonical UPR^mt^ axis described above to decrease the folding load and increase the folding capacity, respectively, in an attempt to overcome mitochondrial protein misfolding. In addition, mitochondria can activate a third and additional UPR^mt^ axis.

## The UPR^mt^ sirtuin axis

Mitochondrial dysfunction often causes a proteotoxic oxidative environment. To counteract this potential source of misfolded proteins, cells can activate the UPR^mt^ sirtuin axis, exerting antioxidant activity. Sirtuins are lysine deacetylases and ADP-ribosyltransferases controlling a wide range of cellular processes, many affecting mitochondrial function [59, 60]. Regulation of metabolism by sirtuins has been associated with longevity and aging [59, 61]. In mammalian cells, there are seven sirtuins (SIRT1–7) with distinct cellular localization and function: (1) SIRT1, 6, and 7 predominantly localize to the nucleus and control the acetylation state of proteins such as histones, PGC1α (a mitochondrial biogenesis factor) and forkhead box O (FOXO) transcription factors [59, 60, 62, 63]; (2) SIRT2 localizes to the cytosol and controls tubulin and PGC1α acetylation [60, 64]; and (3) SIRT3–5 localize to mitochondria and mainly control metabolic processes such as the Krebs cycle and fatty acid oxidation [59, 65]. Strikingly, sirtuins, particularly SIRT1 and SIRT3, have also been shown to be involved in the UPR^mt^.

SIRT1/SIRT 3 had been known to exert an antioxidant effect by controlling the activity and localization of the transcription factor FOXO3A [66, 67]. Deacetylation of FOXO3A by SIRT1/SIRT3 drives FOXO3A localization to the nucleus [68], where it stimulates the transcription of antioxidant enzymes such as the mitochondrial superoxide dismutase 2 (SOD2) and catalase [69, 70]. Strikingly, the same mechanism is triggered by proteotoxic folding stress in the mitochondrial matrix, leading to activation and increased levels of SIRT3 and subsequently eliciting an antioxidant response via FOXO3A deacetylation and the induction of SOD2 and catalase [48, 71]. The observed effects are dependent on the production of ROS and also entail the lipidation of LC3B, induction of several autophagy genes, and increased autophagy rates, suggesting a stimulation of autophagy and/or autophagic flux [48]. These effects were also confirmed by direct sirtuin activation via chemically increasing NAD^+^ levels, thereby causing an elevated mitochondrial antioxidant activity in both *C. elegans* and mammalian cells [72, 73]. Recently, SIRT3 was shown to bind to ATP synthase and to be stimulated upon mitochondrial depolarization via a pH-dependent dissociation from ATP synthase, linking respiratory stress and SIRT3 activity [65]. Additionally, different mitochondrial stresses not related to protein misfolding and directly causing ROS production are also capable of SIRT3 induction, further emphasizing the role of the sirtuin axis as an antioxidant response, but also indicating SIRT3 levels alone cannot serve as a marker for the UPR^mt^ [48]. Importantly, the SIRT3–FOXO3A axis is independent of CHOP, as seen by RNAi-mediated knockdown of CHOP and inhibition of SIRT3 having no effect on the canonical UPR^mt^ transcriptional response [48]. With production of ROS as an ample byproduct of mitochondrial dysfunction, the antioxidant activity of the UPR^mt^ sirtuin axis is likely highly complementary to the canonical UPR^mt^ transcriptional response in securing mitochondrial health.

## The UPR^IMS^–ERα axis

The IMS is separated from the matrix by a membrane forming a distinct compartment in which protein misfolding can occur, leading to a distinct mitochondrial UPR—the UPR^IMS^. Its underlying features have been largely described by the use of endonuclease G (EndoG), an IMS endonuclease released from mitochondria to fragment DNA, causing caspase-independent apoptosis upon conditions such as heat and oxidative stress [74–77]. Expression of mutant EndoG leads to accumulation of misfolded EndoG in the IMS and clustering of mitochondria [78, 79]. This process elicits an IMS UPR (UPR^IMS^) that appears to be independent of the matrix UPR^mt^ and does not cause induction of *CHOP* or *HSP60* [79]. Thus, it does not signal through the ISR or the canonical UPR^mt^ transcriptional response. Instead, its signaling is dependent on estrogen receptor alpha (ERα) and mediated by ROS-dependent phosphorylation of ERα by AKT [79]. Activated ERα then leads to (1) increased nuclear respiratory factor 1 (NRF1) transcript and protein levels, a factor known to regulate proteasome levels [80], the mitochondrial transcription machinery, and thus respiration [81], (2) elevated transcript and protein levels of the IMS protease OMI [79, 82], and (3) an increase in proteasome activity [79]. Together, these effects increase the protein quality control (PQC) system to prevent import and accumulation of (defective) IMS proteins in the IMS [78, 79]. Strikingly, in cells not expressing ERα, misfolding within the IMS leads to induction of *CHOP* and *HSP60* similarly to the effects observed upon inducing matrix protein misfolding [47], suggesting that, upon loss of the IMS PQC machinery and accumulation of misfolded proteins in the IMS, either the canonical UPR^mt^ transcriptional response becomes activated directly by unknown mechanisms, or that the severe accumulation of misfolded IMS proteins causes matrix protein misfolding that activates the canonical UPR^mt^ transcriptional response as an indirect response to perturbed IMS proteostasis. The UPR^IMS^–ERα axis defines a distinct response from the UPR^mt^ axes, attempting to specifically modulate IMS proteostasis to improve folding. Depending on the environment causing protein misfolding, it may act in parallel to the UPR^mt^ axes. UPR^mt^ induction upon UPR^IMS^ failure shows the important role IMS proteostasis exerts on folding in the matrix and suggests possible links between these responses.

## Mitochondrial stress responses

**Fig. 4:**
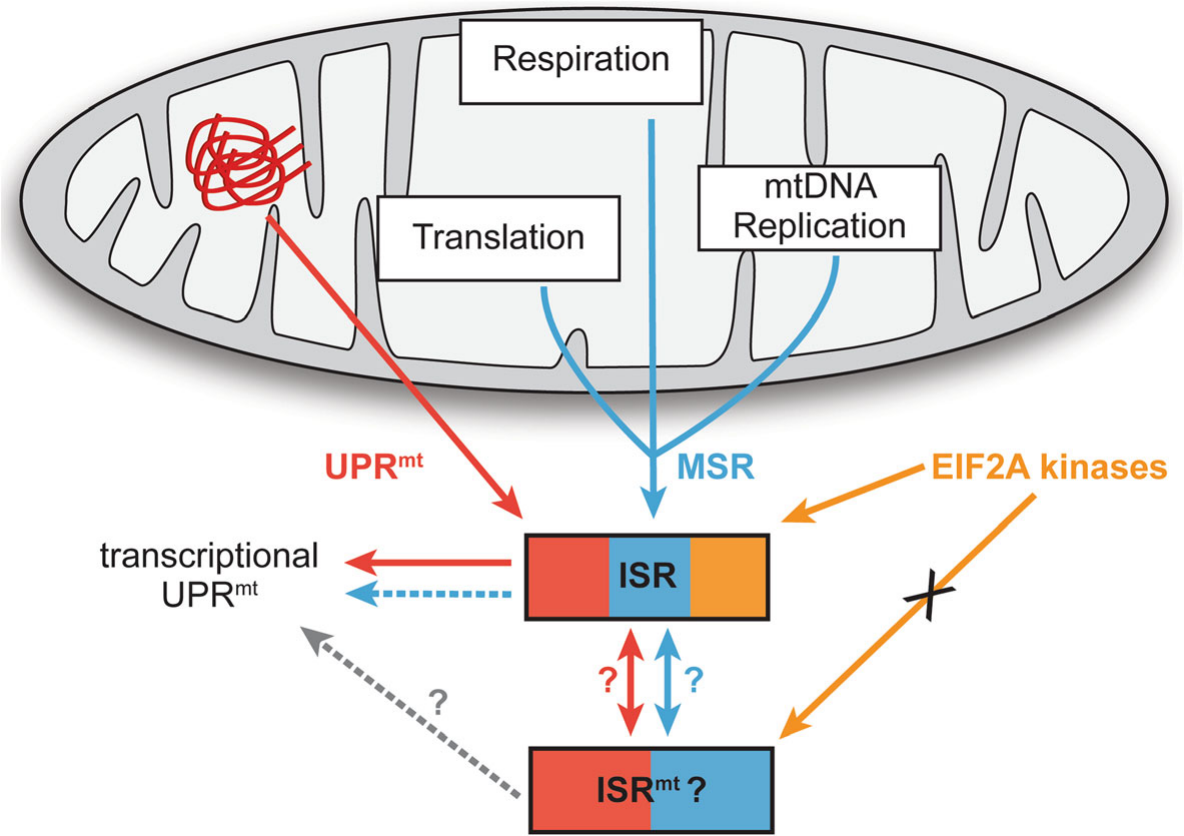
Mitochondrial stress responses. Various mitochondrial stresses, not directly linked to mitochondrial protein misfolding, elicit stress responses that are similar to the UPR^mt^ retrograde signaling and involve the integrated stress response (*ISR*). These stresses affect important aspects of mitochondria, such as respiration, translation, and mtDNA replication and are distinct from the UPR^mt^ transcriptional responses. Recent findings suggest a specific mitochondrial ISR that shares common factors with the ISR but driven by different signaling pathways and eliciting alternative outputs

Importantly, several mitochondrial stress responses (MSRs) are not apparently induced by mitochondrial protein misfolding, but still show a certain degree of similarity to the UPR^mt^ by relying on overlapping pathways (Fig. 4). Of particular importance are the distinct stresses that lead to activation of the ISR and result in activation of specific transcriptional profiles, as mentioned above. In addition to these stresses that all involve induction of ATF4, several other stresses have been studied in detail, which have not been directly associated with protein unfolding, but trigger a MSR and the ISR: (1) mutations in twinkle, a mtDNA helicase, lead to mtDNA deficiencies resulting in respiratory chain deficiency and mitochondrial myopathy [83]. Skeletal muscle of mutant twinkle mice, carrying a dominant duplication of 13 amino acids in twinkle, accumulate mtDNA deletions and show an induction of *Atf4* and *Atf5*, mediated by mTOR activity [84]. This response also activates markers of the canonical UPR^mt^ transcriptional response, suggesting crosstalk with this pathway [84]. However, whether this response is mediated by protein misfolding in the matrix remains unclear. (2) Knockout of the mitochondrial tRNA synthetase *Dars2* in mice causes mitochondrial translation defects and respiratory deficiency without any apparent effects on mitochondrial protein misfolding [85]. DARS2-deficient hearts show upregulation of *Chop*, *Atf4*, and *Atf5*, thereby demonstrating activation of the ISR [85]. Strikingly, the lethality caused by *Dars2* knock-out is alleviated by an additional loss of CLPP without modulating the transcriptional response observed upon Dars2 knock-out [86]. (3) Overexpression of CLPX, the AAA+ ATPase unfoldase lid of the mitochondrial CLPP protease [87], induces a retrograde transcriptional pathway mediated by CHOP through an unknown mechanism [50]. Increasing ClpX levels stimulate the degradation capacity of ClpXP [88]. Thus, an accumulation of non-degraded CLPXP substrates is unlikely, suggesting a signaling pathway not defined by a lack of degradation of CLPP substrates. (4) Knockout of Surf1 in the skeletal muscle of mice causes induction of *CHOP*, *HSP60*, and *LONP* [89, 90]. Surf1 is a complex IV assembly factor and knock-out leads to decreased respiration without apparent effects on mitochondrial protein folding [91]. Thus, the observed effects of *Surf1*^−/−^ are likely mediated by similar mechanisms as observed upon pharmacological ablation of respiration. Strikingly, *Surf1*^−/−^ mice exhibit an increase in mitochondrial number and longevity [89, 91], pointing towards a mechanism of increased robustness due to the defects in respiration. (5) Inhibition of mitochondrial translation leads to GCN2-dependent ISR induction activating *CHOP*, without stimulating chaperonins [92].

Together, these described MSRs are able to induce adaptation mechanisms, largely mediated by retrograde signaling (via the ISR) and transcriptional modulation, and some potentially utilize signaling through the canonical UPR^mt^ transcriptional response (Fig. 4). There appear to be common signaling pathways broadly activated upon mitochondrial stress. To what extent these overlap molecularly with UPR^mt^ signaling and whether some of the described MSRs also involve mitochondrial protein misfolding to activate the UPR^mt^ is an important and challenging question for future research. Due to the current lack of insight and to avoid confusion as to the numerous mitochondrial stresses and responses observed, it is important to distinguish between (1) MSRs and UPR^mt^, depending on the significant/primary involvement of mitochondrial protein misfolding as causative agent, and (2) the UPR^mt^ axes studied by clearly determining and describing the UPR^mt^ axes monitored and activated.

Many of the MSRs signal, at least in part, via the ISR. One of the future challenges is to determine to what extent MSR and UPR^mt^ signaling through the ISR is overlapping with the activation of the ISR by EIF2A kinases. Some significant differences between classic activation of the ISR by EIF2A kinases and activation by mitochondrial stress have already been described, leading to the proposal of a specific mitochondrial ISR (ISR^mt^), distinct from the canonical ISR [84]. Further research will be required to determine the role of such a ISR^mt^ in the UPR^mt^ and to determine the temporal control and cross-activation of the ISR^mt^ and the different UPR^mt^ axes in response to mitochondrial misfolding stress.

## Summary and future outlook

Various conditions, where mitochondrial protein misfolding is the primary cause of mitochondrial stress, have been shown to activate axes of the UPR^mt^. Strikingly, inhibition of mitochondrial HSP90 to induce protein misfolding and the UPR^mt^ has been recently explored as a potent therapeutic strategy to target cancer [93, 94]. The different effects elicited by the different UPR^mt^ axes demonstrate that the UPR^mt^ is a multi-pronged response modulating several aspects of mitochondrial proteostasis in an attempt to alleviate folding stress (Fig. 2). The UPR^mt^ axes are distinct with specific sets of defining factors, allowing and encouraging researchers to clearly define, describe, and validate activation of the proposed axis studied. The different UPR^mt^ axes are pro-survival and attempt to maintain mitochondria. However, there must also be destructive pathways activated upon failure to restore proteostasis. Strikingly, there have now been numerous reports of mitochondrial protein misfolding triggering LC3B lipidation, induction of autophagy genes, and mitophagy [41, 44, 48, 94, 95], suggesting that autophagy in general and the selective degradation of damaged mitochondria via this route might play a significant role in the UPR^mt^. However, substantiated evidence proving a causative relation between mitochondrial protein misfolding and autophagy is still lacking, and the same applies to insight into the mechanisms involved. Thus, the relationship between non-selective autophagy, mitophagy, and the UPR^mt^ will require further investigation. It is tempting to speculate that these studies will reveal autophagy pathways forming an additional UPR^mt^ axis that might initiate upon more severe or prolonged mitochondrial protein misfolding, when the described UPR^mt^ axes fail to restore proteostasis, or are overburdened.
